# Assessment of recently developed blood gas analysers: a multicentre evaluation

**DOI:** 10.1155/S1463924689000519

**Published:** 1989

**Authors:** B. Gouget, Y. Gourmelin, A. Feuillu, F. Blanchet, B. Capolaghi, M. Lagente, G. Lardet, J. C. Manceaux, A. Truchaud

**Affiliations:** Biochemistry Laboratory Centre Hospitalier Général, BP 218 Meaux F 77108 France

## Abstract

Providing guidelines for testing expected inaccuracy and imprecision is still a matter under debate. The Expert Panel of the French Society of Clinical Chemistry has developed a protocol, which was based on a comparative multi-centre evaluation of four instruments: the Ciba-Corning 278, the Instrumentation Laboratory 1306, the Nova SP 5 and the ABL 330. The purpose was to evaluate the analytical performance and efficiency of the analysers. Another aim was to design a valid approach for evaluating any new system. As buffered aqueous solutions and fluorocarbon emulsions give only partial information, tonometered blood was used at different levels of gas mixture, even though it is both difficult and time-consuming. Comparisons have been established on patients' blood samples with the analysers currently used in the evaluation sites. The tests showed that the four analysers have the same degree of precision, and interinstrument comparisons demonstrated a very high degree of reliability.

This analysis emphasizes that the evaluation of instruments for pH and blood gas analysis is neither easy nor is it often done, mainly due to the choice of a quality-control material and the lability of the measured parameters.


Journal of Automatic Chemistry Vol. 11, No. 6 (November–December 1989), pp. 266–272

### Assessment of recently developed blood gas analysers: a multicentre evaluation

**B. Gouget,† Y. Gourmelin,† A. Feuillu,‡ F. Blanchet,‡ B. Capolaghi,‡ M. Lagente,‡ G. Lardet,‡ J. C. Manceaux‡ and A. Truchaud†**

*† Biochemistry Laboratory, Centre Hospitalier Général, F 77108 Meaux, BP 218, France*

*‡ Expert Panel of Instrumentation of the Société Française de Biologie Clinique. (Chairman: Dr Grafmeyer, Biochemistry Laboratory, CHR de la Croix Rousse, F 69317 Lyon Cédex 04, France)*

*Providing guidelines for testing expected inaccuracy and imprecision is still a matter under debate. The Expert Panel of the French Society of Clinical Chemistry has developed a protocol, which was based on a comparative multi-centre evaluation of four instruments: the Ciba-Corning 278, the Instrumentation Laboratory 1306, the Nova SP 5 and the ABL 330. The purpose was to evaluate the analytical performance and efficiency of the analysers. Another aim was to design a valid approach for evaluating any new system. As buffered aqueous solutions and fluorocarbon emulsions give only partial information, tonometered blood was used at different levels of gas mixture, even though it is both difficult and time-consuming. Comparisons have been established on patients' blood samples with the analysers currently used in the evaluation sites. The tests showed that the four analysers have the same degree of precision, and inter-instrument comparisons demonstrated a very high degree of reliability.*

*This analysis emphasizes that the evaluation of instruments for pH and blood gas analysis is neither easy nor is it often done, mainly due to the choice of a quality-control material and the lability of the measured parameters.*

### Introduction

Modern pH and blood gas analysers measure pH, $pCO_2$ and $pO_2$ by electrochemical methods. Built-in microprocessors have not only improved automation and signal processing but also the calculation of parameters for oxygen status and acid base balance [1]. A multi-centre evaluation was organized by the French Society of Clinical Chemistry in order to compare the analytical performance and the practicability of four recent models so that guidelines could be given on the choice of equipment. The study also looked at quality control in bood gas analysis.

### Materials and methods

### *Instrumentation*

New models from four manufacturers were tested: Ciba-Corning 278 (USA); IL 1306 Instrumentation Laboratory (USA); Stat Profile 5 Nova Biomedical (USA); ABL 330 Radiometer (Denmark). Two instruments of the same type located in two different places were evaluated. In each site, the usual routine analyser was used for instrument comparison shown below:

| Site | Tested instrument | Comparison instrument |
|---|---|---|
| 1 | Ciba Corning 278 | ABL 3 Radiometer |
| 2 | Ciba Corning 278 | Ciba Corning 178 |
| 3 | IL 1306 | Ciba Corning 178 |
| 4 | IL 1306 | Ciba Corning 178 |
| 5 | NOVA SP 5 | ABL 3 Radiometer |
| 6 | NOVA SP 5 | Ciba Corning 178 |
| 7 | ABL 330 Radiometer | Ciba Corning 178 |
| 8 | ABL 330 Radiometer | ABL 300 Radiometer |

### *Materials*

#### *Solutions*

Phosphate buffer solutions, at pH = 7·384 and 6·838, were prepared in line with the National Bureau of Standards recommendation for standard reference material for pH measurement [2].

Commercial aqueous control solutions were used at three levels (acidosis, normal, alkalosis). Brands of instruments and solutions were as recommended: Certain for Corning, Nova SP control for Nova SP 5 and Qualicheck for Radiometer.

Fluorocarbon emulsions (ABC, IL-Fisher) were used for IL 1306.

#### *Tonometry*

Tonometry was performed according to the recommendations of the proposed IFCC reference method for tonometry of blood [3] on the Laue bulb tonometer (L. Eschweiller & Co., Kiel, FR Germany) and the IL 237 tonometer (Instrumentation Laboratory, Inc. Lexington, Massachusetts, USA) with a gas mixture of known composition ($pO_2$ and $pCO_2$).

#### *Blood samples*

Fresh heparinized blood was drawn from healthy donors for tonometry and from hospitalized patients for comparison studies.

### Protocol

### *pH accuracy*

Measurements should be ideally performed on whole blood but stabilization of this medium is critical [4]. Thus the two phosphate buffers were run in triplicate over five days on both comparison and tested instruments.

### *Precision study*

*Within-run precision* was estimated with aqueous solutions or fluorocarbon emulsions and with two levels of

0142–0453/90 $3.00 © 1990 Taylor & Francis Ltd.

tonometry L1:$CO_2$ ≈ 5%, $O_2$ ≈ 12%; $L_2$:$CO_2$ ≈ 12%, $O_2$ ≈ 5·5%). For each sequence, six measurements were performed daily on the three first and last days of the evaluation.

*Day-to-day precision*: The same solutions were used but tonometries were slightly different (L1:$CO_2$ ≈ 5%, $O_2$ ≈ 21%; L2:$CO_2$ ≈ 12%, $O_2$ ≈ 5·5%). Measurements were run daily for 20 days, after preliminary calibration of the instruments.

*Drift* was checked using tonometered blood tested immediately after a calibration and before the subsequent one.

*Linearity* was assessed using three successive measurements of tonometered whole blood containing $O_2$ from 0 to 95% and $CO_2$ from 2 to 15%. The sequence was repeated three times during the evaluation period.

*Carry-over* was looked for using two different tonometered bloods with high (H) and low (L) percentages of $O_2$ and $CO_2$ respectively, following the sequence: LLL-HHH-LLL. (H:$CO_2$ ≈ 12%, $O_2$ ≈ 55%; L:$CO_2$ ≈ 3%, $O_2$ ≈ 5·5%).

*Inter-instrument comparisons*: In each site 100 samples from patients were measured simultaneously on the tested and comparison instruments. Values for the three parameters covered the pathophysiological range as indicated below:

| Repartition of the values (%) | 20 | 60 | 20 |
|---|---|---|---|
| pH | <7·30 | 7·30–7·50 | >7·50 |
| $pCO_2$ mmHg | <30 | 30–60 | >60 |
| $pO_2$ mmHg | <50 | 50–200 | >200 |

*Practicability*

Special attention was paid to quantification of the most important specifications of the four models. All the evaluating sites were provided with guidelines in order to translate 'subjective' appreciation into scales for the different items, focusing on handling of instruments, microprocessor flexibility, reagents and disposable materials, sample volume, throughput, the degree of skill and time required of the users, maintenance and repair procedures, reliability of the software diagnostic messages.

### Results

*pH accuracy*: For each model data for pH ranged ±0·02 pH from the assigned value of National Bureau of Standards buffers.

*Precision study*

*Within-run precision* results are reported in table 1. Data from only one site are reported and a representative sequence is given for each model and each case. It is noteworthy that the most scattered sequences were obtained on $pO_2$ determinations with aqueous buffered solutions or fluorocarbon emulsions. Tonometry sequences yield uniform results for $pO_2$ and $pCO_2$, whatever the levels. The maximal variation measured within a single series never exceeded 2 mmHg for $pCO_2$ and 1·5 mmHg for $pO_2$, regardless of level or model. All the measured values were very close to the theoretical values.

*Day-to-day precision* data for the three parameters are shown in table 2. With both aqueous solutions and fluorocarbon emulsions, day-to-day precision was uniform between the instruments. As for tonometry, results were very close to the assigned values.

*Linearity*: Linearity for $pCO_2$ was verified from 15 to 80 mmHg on the NOVA SP 5, up to 100 mmHg on the IL 1306; up to 110 mmHg on the ABL 330; and up to 140 mmHg on the Ciba Corning 278. These different values were the maximal tested values by tonometry in the different sites. The instruments exhibited a tendency to underestimate $pO_2$ values by −4%, above 700 mmHg for the Ciba Corning 278; by −2·5% above 400 mmHg on the IL 1306; by −4% above 550 mmHg on the NOVA SP 5; and by −3% above 500 mmHg on the ABL 330.

Neither *drift* nor *carry-over* were observed in any of the instruments.

*Inter-instrument comparisons*

Table 3 summarizes the individual results.

*pH*: Only the IL 1306 instruments yielded pH results slightly lower than the comparison instrument (Ciba Corning 178).

*$pCO_2$*: The ABL 330 Radiometer instruments gave values identical to the comparison ones (ABL 300) between 15 and 90 mmHg. In one site the Nova SP5 exhibited excellent results but in the other site the values were lower than the comparison one (Ciba-Corning 178) above 60 mmHg. The two IL 1306 instruments gave similar results, identical to the comparison analyser (Ciba-Corning 178) up to 70 mmHg. In one site the Ciba-Corning 278 gave a slight variation of the values, never exceeding +3 mmHg.

*$pO_2$*: ABL 330 Radiometer demonstrated discrepant results. In one site, they were very similar to the comparison instrument, but in the other site, $pO_2$ values were underestimated by about 15 mmHg; the same observations were made for the Ciba Corning 278. The IL 1306 instruments gave more scattered values and these were generally lower than obtained on the comparison instruments above 200 mmHg. Results obtained on the NOVA SP 5 were similar to the comparison instrument in one site but more scattered in the other site.

*Practicability*: The systems are easy to operate. They are not bulky, they are quick, they are available 24 h a day and require only small volumes of whole blood. Membrane replacement procedures have been facilitated by the new electrode design.

As an example, the Ciba Corning instrument is equipped with maintenance-free electrodes and so remembraning is unnecessary. When an electrode needs to be replaced, the procedure is quick and easy: simply pull out the old electrode and slide in the new one. The software flexibility

*Table 1. Within-run precision for one sequence (N = 6).*
(a) Aqueous solutions and fluorocarbon emulsions.

| pH | Target value | Mean | S.D. | Target value | Mean | S.D. | Target value | Mean | S.D. |
|---|---|---|---|---|---|---|---|---|---|
| CC 278 | 7·147 ± 0·02 | 7·158 | 0·03 | 7·410 ± 0·02 | 7·397 | 0·03 | 7·611 ± 0·02 | 7·611 | 0·005 |
| IL 306 | 7·210 ± 0·02 | 7·211 | 0·03 | 7·392 ± 0·02 | 7·389 | 0·02 | 7·560 ± 0·02 | 7·574 | 0·002 |
| SP 5 | 7·210 ± 0·02 | 7·216 | 0·03 | 7·397 ± 0·02 | 7·409 | 0·02 | 7·470 ± 0·02 | 7·584 | 0·003 |
| ABL 330 | 7·120 ± 0·015 | 7·123 | 0·02 | 7·380 ± 0·015 | 7·374 | 0·02 | 7·617 ± 0·015 | 7·616 | 0·002 |
| $pCO_2$ (mmHg) | Target value | Mean | C.V. % | Target value | Mean | C.V. % | Target value | Mean | C.V. % |
| CC 278 | 22 ± 2 | 22·5 | 1·7 | 40·6 ± 3 | 43·5 | 1·1 | 61·6 ± 5 | 60·0 | 2·0 |
| IL 1306 | 24 ± 3 | 23·4 | 1·8 | 42 ± 3 | 42·9 | 1·1 | 66 ± 5 | 69·4 | 1·2 |
| SP 5 | 22 ± 2 | 20·8 | 0·8 | 42 ± 3 | 39·9 | 1·0 | 62 ± 5 | 62·2 | 1·4 |
| ABL 330 | 18·9 ± 2 | 18·3 | 1·0 | 40 ± 3 | 39·8 | 0·9 | 61·5 ± 5 | 59·8 | 0·9 |
| $pO_2$ (mmHg) | Target value | Mean | C.V. % | Target value | Mean | C.V. % | Target value | Mean | C.V. % |
| CC 278 | 68 ± 6 | 70·5 | 1·3 | 106 ± 6 | 105·0 | 1·6 | 134 ± 6 | 132·0 | 1·0 |
| IL 306 | 56 ± 6 | 59·0 | 1·3 | 98 ± 6 | 99·3 | 1·2 | 144 ± 6 | 141·6 | 0·9 |
| SP 5 | 58 ± 6 | 56·7 | 1·9 | 98 ± 6 | 99·5 | 1·4 | 141 ± 6 | 138·9 | 0·5 |
| ABL 330 | 50 ± 8 | 56·3 | 2·8 | 106 ± 6 | 106·8 | 1·0 | 182·5 ± 7·5 | 181·1 | 0·9 |

*(b) Blood tonometry.*

| $pCO_2$ (mmHg) | Assigned value | Mean | C.V. % | Assigned value | Mean | C.V. % |
|---|---|---|---|---|---|---|
| CC 278 | 35 | 35·2 | 0·8 | 86 | 87·1 | 1·6 |
| IL 1306 | 35 | 35·7 | 0·8 | 85 | 85·6 | 0·8 |
| SP 5 | 35 | 35·7 | 0·7 | 85 | 86·2 | 0·6 |
| ABL 330 | 40 | 39·8 | 0·5 | 85 | 84·6 | 0·5 |
| $pO_2$ (mmHg) | | | | | | |
| CC 278 | 40 | 41 | 0·9 | 85 | 86 | 0·9 |
| IL 1306 | 40 | 40·8 | 1·0 | 85 | 85·7 | 0·7 |
| SP 5 | 45 | 44·2 | 1·4 | 85 | 84·1 | 0·9 |
| ABL 330 | 45 | 45·5 | 0·9 | 85 | 85·5 | 0·7 |

provides many advantages related to patient sample calibration, quality-control and printing. Information on software is given in table 4. On the last generation instruments, repair procedures are simple since major systems modules are easily accessible. Different instruments can be connected to a panel of interfaces of other peripheral or central computerized devices [5].

### Discussion

The overall tests of this evaluation demonstrated the uniformity of the four instruments in terms of pH measurement, since each gave reliable results in the precision study and inter-instrument comparisons.

For blood gas analysis, all the instruments showed acceptable imprecision. Variability of the values changes mainly with the type of quality control solution, rather than with the instrument model.

Differences in values of about 0·025 UpH were observed between the IL 1306 and the Ciba Corning 178, as already reported by the College of American Pathologists. The pH measurement systems differ between the two manufacturers: the cells for pH measurement consist of a glass electrode and a reference electrode. However, the electrodes have been designed with various geometrical designs and different kinds of glass materials. These routine methods differ perceptibly from the reference method given by the International Federation of Clinical Chemistry for pH measurement in blood [4]. The routine pH systems are usually calibrated with secondary calibration solutions. They often provide highly precise, but not necessarily accurate, pH data because of variations of pH electrode systems, liquid/liquid junctions, calibration and measurement procedures. It is quite impossible to decide on which pH electrode is more accurate between IL (with lower systematic pH values) and Ciba Corning. However, inter-instrument comparisons showed that pH and $pCO_2$ were two parameters with nearly uniform measurements. When discrepancies occurred, preanalytical errors were probably responsible.

Imprecision and inaccuracy were more difficult to appreciate for $pO_2$ than for $pCO_2$, mainly due to the high

*Table 2. Day-to-day precision (N = 20)*
*(a) Aqueous solution and fluorocarbon emulsions.*

| pH | Mean | S.D. | Mean | S.D. | Mean | S.D. |
|---|---|---|---|---|---|---|
| CC 278 | 7·150 | 0·003 | 7·396 | 0·004 | 7·618 | 0·005 |
| IL 1306 | 7·207 | 0·006 | 7·388 | 0·004 | 7·578 | 0·007 |
| SP 5 | 7·211 | 0·007 | 7·402 | 0·010 | 7·580 | 0·006 |
| ABL 330 | 7·121 | 0·003 | 7·377 | 0·002 | 7·615 | 0·002 |

| $pCO_2$ (mmHg) | Mean | C.V. % | Mean | C.V. % | Mean | C.V. % |
|---|---|---|---|---|---|---|
| CC 278 | 23·4 | 2·9 | 44·2 | 3·3 | 60·4 | 2·3 |
| IL 1306 | 24·5 | 2·1 | 40·6 | 1·8 | 64·6 | 1·4 |
| SP 5 | 21·8 | 3·6 | 41·0 | 2·9 | 61·2 | 3·4 |
| ABL 330 | 18·3 | 1·5 | 40·7 | 1·0 | 60·8 | 0·9 |

| $pO_2$ (mmHg) | Mean | C.V. % | Mean | C.V. % | Mean | C.V. % |
|---|---|---|---|---|---|---|
| CC 278 | 69·1 | 2·2 | 105·5 | 1·8 | 132.2 | 1·2 |
| IL 1306 | 60·2 | 2·4 | 102·3 | 1·3 | 141·4 | 0·6 |
| SP 5 | 59·4 | 4·2 | 94·9 | 2·2 | 140·8 | 2·0 |
| ABL 330 | 50·8 | 3·8 | 103·4 | 1·7 | 176·8 | 1·5 |

(b) Blood tonometry.

| $pCO_2$ (mmHg) | Assigned value | Mean | C.V. % | Assigned value | Mean | C.V. % |
|---|---|---|---|---|---|---|
| CC 278 | 36·0 | 35·7 | 3·9 | 84·4 | 85·6 | 2·9 |
| IL 1306 | 31·9 | 32·8 | 1·8 | 90·4 | 91·4 | 2·5 |
| SP 5 | 31·3 | 31·4 | 4·8 | 84·8 | 83·2 | 3·8 |
| ABL 330 | 35·1 | 35·3 | 3·7 | 85·8 | 83·7 | 2·2 |

| $pO_2$ (mmHg) | Assigned value | Mean | C.V. % | Assigned value | Mean | C.V. % |
|---|---|---|---|---|---|---|
| CC 278 | 39·9 | 41·7 | 1·4 | 159·0 | 157·9 | 1·5 |
| IL 1306 | 46·1 | 43·8 | 1·0 | 144·0 | 143·4 | 3·6 |
| SP 5 | 43·9 | 43·9 | 2·5 | 142·3 | 141·8 | 1·1 |
| ABL 330 | 42·8 | 41·8 | 1·9 | 140·4 | 139·7 | 0·7 |

sensitivity of this parameter, the non-linearity of oxyhaemoglobin saturation curve, the technologies involved, and the difficulties assembling the equipment to study the problem. In addition, preanalytical errors in collecting and handling specimens can have a significant impact on $pO_2$ measurement. As accuracy is critical for $pO_2$, it was decided to evaluate the analytical performance of the instruments under 'experimental', rather than 'daily routine' conditions. The overall mean values obtained with commercial quality-control solutions in this study were not necessarily the 'target' values, but generally overlapped those indicated by manufacturers, which varied according to brand and type [6 and 7]. Commercially available quality-control materials have limitations: their physical and chemical properties often do not match those of blood. With these controls, a substantial inter-instrument difference could not be clearly demonstrated. Fluorocarbon emulsions did not demonstrate benefits in comparison with aqueous solutions [8]. This parallels to the larger question of whether the instrument differences found using quality-control ampoules are likely to predict similar differences if clinical samples of whole blood were to be measured.

Tonometered blood is ideal for examining inter-instrument bias in $pO_2$, but when it is necessary to use more than one tonometer and more than a single source of tonometry gas, the variability of the blood gas measurement of $pO_2$ increases. Thus the analysis and interpretation of systematic bias becomes more difficult. Whole-blood tonometry allows the accuracy and the precision of the instruments to be evaluated [9]. However, this method requires knowledge of the composition of the gas mixture, control of the tonometer temperature and of the time for complete equilibration. No mishandling should occur during the transfer of the equilibrated sample from the tonometer to the instrument [2]. Not only is the technique time-consuming but also the equipment is not widely available in France.

$pO_2$ inter-instrument comparison with blood specimens identified the difficulty in maintaining the validity of $pO_2$ measurement. There are instrument differences in calibration methods for $pO_2$ electrodes and measuring chamber size, measuring chamber content prior to sample introduction, sample introduction technique, sample size, sample warming, analysis time, and electrode signal processing, all of which can contribute to model-specific differences. Whether the inter-instrument differences in $pO_2$ are real is still much discussed [10–12].

The linearity study showed that the $pO_2$ responses of the sensors were in line with the manufacturers' specifications. This indicates that model specific algorithms to correct design and imperfections are valid. In fact, the discrepancies observed for the high values of $pO_2$ are not really clinically relevant.

In this comparative study, the basic models were tested for each brand (see table 5). Thus three out of four analysers measured only pH and $pO_2$, $pCO_2$. NOVA SP 5, chosen in agreement with the manufacturer, is a good example of a multichannel analyser combining determinations of other analytes. The appearance of combined electrochemical sensors for electrolytes, analytes, pH and blood gases have raised new issues in sensor calibration, sample collection and handling. Phosphate buffers, developed for pH calibration, interfere with the activities of $Na^+$, $K^+$, $Ca^{2+}$ in calibrating solutions. On the other hand, organic buffers, which do not affect these ions activities, lead to a pH bias on sample measurement [13].

Besides the quantification of the analytical performance of the analysers, this protocol draws users' attention to appropriate indicators of functioning for each system, and the difficulty in obtaining reliable data with respect to the various quality-control materials. The quality-control materials which are available today are not ideal. Aqueous materials have such advantages as a long shelf-life and being prepared in ampoules which are ready to use; their disadvantage is poor oxygen buffering [14]. Even fluorocarbon emulsions have an oxygen buffering capacity sufficient for the normal $pO_2$ level only, they are not sensitive enough to temperature, especially for $pO_2$

*Table 3. Inter-instrument comparisons: equation of allometry lines and r (coefficient of correlation).*

| | Site | *N* | pH Equations of allometry lines | pH *r* | $pCO_2$ Equations of allometry lines | $pCO_2$ *r* | $pO_2$ Equations of allometry lines | $pO_2$ *r* |
|---|---|---|---|---|---|---|---|---|
| CC 278 | 1 | 97 | $y = 1{\cdot}00x - 0{\cdot}02$ | 0·995 | $y = 1{\cdot}02x + 0{\cdot}06$ | 0·986 | $y = 0{\cdot}88x + 8{\cdot}18$ | 0·992 |
| | 2 | 97 | $y = 1{\cdot}01x - 0{\cdot}08$ | 0·997 | $y = 1{\cdot}05x - 2{\cdot}66$ | 0·995 | $y = 0{\cdot}99x + 2{\cdot}25$ | 0·998 |
| IL 1306 | 3 | 136 | $y = 0{\cdot}95x + 0{\cdot}33$ | 0·988 | $y = 0{\cdot}94x + 1{\cdot}81$ | 0·994 | $y = 0{\cdot}91x + 8{\cdot}64$ | 0·990 |
| | 4 | 96 | $y = 0{\cdot}99x + 0{\cdot}04$ | 0·993 | $y = 0{\cdot}95x + 0{\cdot}72$ | 0·989 | $y = 0{\cdot}89x + 5{\cdot}91$ | 0·997 |
| SP 5 | 5 | 115 | $y = 0{\cdot}97x + 0{\cdot}18$ | 0·997 | $y = 0{\cdot}88x + 2{\cdot}92$ | 0·996 | $y = 1{\cdot}02x + 2{\cdot}19$ | 0·998 |
| | 6 | 118 | $y = 0{\cdot}98x + 0{\cdot}11$ | 0·994 | $y = 0{\cdot}97x + 1{\cdot}44$ | 0·993 | $y = 0{\cdot}98x + 0{\cdot}72$ | 0·999 |
| ABL 330 | 7 | 145 | $y = 0{\cdot}99x + 0{\cdot}04$ | 1 | $y = 1{\cdot}00x - 0{\cdot}29$ | 0·999 | $y = 0{\cdot}98x + 1{\cdot}49$ | 1 |
| | 8 | 103 | $y = 0{\cdot}99x + 0{\cdot}07$ | 0·991 | $y = 0{\cdot}97x + 1{\cdot}19$ | 0·997 | $y = 0{\cdot}94x + 4{\cdot}58$ | 0·996 |

[15]. Stroma-free haemoglobin solutions, with a plasma-like composition, behave like fresh whole-blood in terms of oxyen affinity. Attention has been given to the possibility of preparing such solutions with low, normal and high electrolyte values, but one can expect problems arising with the different ions activities. However, this type of solution is the most suitable $pO_2$ and haemoglobinometry [16].

Preanalytical conditions also need to be considered [17, 18]. Measurements on whole blood enable extracellular determinations of the parameters with the multichannel analysers. Several processing steps, such as centrifugation, are eliminated. Blood gas analysis must be performed rapidly after sample collection for reliable data. However, storage temperature of the samples will affect measurement of glucose or potassium. Specimens are stable for blood gas analysis for two hours when maintained at about 1 °C, reducing the glycolytic effect, but potassium increased significantly under the same conditions [19]. A compromise can be suggested: if the sample is measured within 10 min it can be maintained at

*Table 4. Practicability of the tested analysers.*

| | Corning 278 | IL 1306 | Nova SP 5 | ABL 330 |
|---|---|---|---|---|
| Flexibility of the functions | | | | |
| Relevance of the information: | ++++ | ++++ | ++++ | ++++ |
| Access to the menu: | +++ | ++++ | ++++ | +++ |
| Display of the results: | ++++ | ++++ | ++++ | ++++ |
| ++++ Excellent | | | | |
| +++ Good | | | | |
| Visibility of the measuring chamber: | | | | |
| 0 Not visible | | | | |
| +++ Total end | | | | |
| ++++ Total, lighted | ++++ | ++++ | +++ | 0 |
| Introduction and volume of the sample: | | | | |
| ++++ Very easy, asp/inj | ++++ | ++++ | +++ | ++++ |
| +++ Easy, asp. | | | | |
| Throughput (/h): | | | | |
| ++ <30 | | | | |
| +++ =30 | +++ | +++ | ++++ | ++ |
| ++++ >30 | | | | |
| Maintenance: | | | | |
| Overall: | ++++ | ++++ | ++++ | ++++ |
| Electrodes: | ++++ | +++ | +++ | +++ |
| Troubleshooting: | +++ | ++++ | ++++ | ++ |
| ++++ Very easy | | | | |
| +++ Easy | | | | |
| ++ Not easy | | | | |
| Reagents packaging: | | | | |
| ++++ Excellent | ++++ | ++++ | +++ | ++++ |
| +++ Very good | | | | |

*Table 5. Commercially available analysers.*

| Manufacturers | Models | Parameters |
|---|---|---|
| AVL | AVL 939 | pH, $pO_2$, $pCO_2$ |
| (Austria) | AVL 990 | pH, $pO_2$, $pCO_2$ |
| | AVL 995 | pH, $pO_2$, $pCO_2$ |
| Ciba-Corning | 170 | pH, $pO_2$, $pCO_2$ |
| (USA) | 178 | pH, $pO_2$, $pCO_2$ |
| | 278 | pH, $pO_2$, $pCO_2$ |
| | 280 | pH, $pO_2$, $pCO_2$, Hb |
| | 288 | pH, $pO_2$, $pCO_2$, Hb, $Na^+$, $K^+$, $Ca^{2+}$, ou $Cl^-$ |
| Instrumentation | IL 1304 | pH, $pO_2$, $pCO_2$ |
| Laboratory | IL 1306 | pH, $pO_2$, $pCO_2$ |
| (USA) | BGE | pH, $pO_2$, $pCO_2$, $Na^+$, $K^+$, Hte, $Ca^{2+}$ |
| Nova Biomedical | Stat profile | |
| (USA) | 1 | pH, $pO_2$, $pCO_2$, $Na^+$, $K^+$, $Ca^{2+}$, Hte |
| | 2 | pH, $pO_2$, $pCO_2$, $Na^+$, $K^+$, Hte |
| | 3 | pH, $pO_2$, $pCO_2$ |
| | 4 | pH, $pO_2$, $pCO_2$, $Na^+$, $K^+$, $Cl^-$, $Ca^{2+}$, Hte |
| | 5 | pH, $pO_2$, $pCO_2$, $Na^+$, $K^+$, $Cl^-$, $Ca^{2+}$, Hte glucose |
| Radiometer | ABL 30 | pH, $pO_2$, $pCO_2$ |
| (Denmark) | ABL 330 | pH, $pO_2$, $pCO_2$ |
| | ABL 300 | pH, $pO_2$, $pCO_2$, Hb |
| | ABL 4 | pH, $pO_2$, $pCO_2$, Hb, $K^+$ |
| | ABL 500 | pH, $pO_2$, $pCO_2$ |

room temperature; if it is measured within 30 min, it should be chilled; an extended delay is in any case not in agreement with the AACC recommendations.

Finally, the choice of anticoagulant is critical when a $Ca^{2+}$ result is needed. Anticoagulant containing calcium or citrate, as well as heparin, will afect ionized calcium values. Calcium titrated heparinate is the best means to minimize calcium chelation, either as a solution in glass ampoule or as a dry preparation in syringe or capillary tube [20–22].

These multichannel analysers offer many advantages, but the question is still open as to the most efficient association of the parameters [23]. The answer depends on the total workload, type and proportion of tests usually ordered, and staffing.

### Conclusion

The modern automated pH and blood gas instruments, with built-in micro-processors, have become a standard equipment in most clinical laboratories or intensive care units and remain the reference technique. The supplementary help of computer programs means that optimal information can be extracted from various and complex data. Although these types of sensors and analysers have proved to be efficient, another field of analytical research is the development of new sensors; the ultimate goal is continuous and non-invasive monitoring of pH and other blood gas parameters.

### Acknowledgements

This work is part of the French co-ordinated evaluation of pH and blood gas analysers organized by The Commission Instrumentation de la Société Française de Biologie Clinique (Chairman: D. Grafmeyer), and with the support of the Centre d'Etudes des Réactifs et Matériels utilisés en Analyses Biologiques (C.E.R.-M.A.B., Documents 88124, 88125, 88126 and 88127).

### References

1. Siggaard-Andersen, O., Wimberley, P. D., Fogh-Andersen, N., and Gothgen, I. H., *Scandinavian Journal of Clinical Laboratory Investigation,* **48** (1988), Suppl. 189, 7.
2. Durst, R. A., *Standard Reference Materials. Standardization of pH Measurements* (National Bureau of Standards, Washington D.C., Special publication 260 55 NBS).
3. Maas, A. H. J. (Ed.), *Journal of Clinical Chemistry and Clinical Biochemistry,* **27** (1989), 403.
4. Maas, A. H. J., Weisberg, H. F., Burnett, R. W., Mueller Plathe, O., Wiberley, P. D., Zijlstra, W. G., Durst, R. A., and Siggaard-Andersen, O., *Clinica Chimica Acta,* **165** (1987), 97.
5. Berg, E., *Scandinavian Journal of Clinical Laboratory Investigation,* **47** (1987), Suppl. 188, 55.
6. Hansen, J. E., Clausen, J. L., Levy, S. E., Mohler, J. G., and Van Kessel, A. L., *Chest,* **89** (1986), 214.
7. Leary, E. T., Graham, G., and Kenny, M. A., *Clinical Chemistry,* **26** (1980), 1309.
8. Gouget, B., Gourmelin, Y., Feuillu, A., and Truchaud, A., *Revue Européenne de Biotechnologie Biomédicale,* **11** (1989).
9. Van Kessel, A. L., Eichhorn, J. H., Clausen, J. L., Stone, M. E., Rotman, H. H., and Crapo, R. O., *Chest,* **92** (1987), 418.
10. Fallon, K. D., Why do different blood gas systems give different answers? *ATS blood gas proficiency program 1983,* **1** (1983), 2.
11. Hansen, J. E., and Feil, M. C., *Chest,* **94** (1988), 49.
12. Eichhorn, J. H., *Chest,* **94** (1988), 1.
13. D'Orazio, P., *Clinical Chemistry,* **35** (1989), 1232.
14. Maas, A. H. J., Veefkind, A. H., Van den Camp, R. A. M., Teunissen, A. J., Winckers, E. K. A., and Jansen, A. P., *Clinical Chemistry,* **23** (1977), 1718.
15. Sprokholt, R., Van Ooik, S., Van den Camp, R. A. M., Bouma, B. N., Zijlstra, W. G., and Maas, A. H. J. *Scandinavian Journal of Clinical Laboratory Investigation,* **47** (1987), Suppl. 188, 69.
16. Sprokholt, R., Van Ooik, S., Van den Camp, R. M., Bouma, B. N., Zijlstra, W. G., and Maas, A. H. J., *Scandinavian Journal of Clinical Laboratory Investigation,* **47** (1987), Suppl. 188, 83.
17. Moran, R. F., and Grenier, R. E., Effects of standard blood gas transport and storage conditions on electrolytes results with observations on reported hemoglobin measurement anomalies. In *Proceedings of the 10th EWGISE Meeting: Methodology and Clinical Applications of Ion Selective Electrodes,* Maas, A. H. J., Buckley, B. M., Manzoni, A., Moran, R., Siggaard-Andersen, O., and Sprokholt, R. (Eds) (Stresa 1988, Elinkwijk Printing Company, Utrecht, The Netherlands, 1989), p. 57.

18. Eichhorn, J. H., Moran, R. F., and Cormier, A. D., Blood gas preanalytical considerations: specimen collection, calibration, and controls. National Committee for Clinical Laboratory Standards (C27), 1988.
19. Fleisher, M., Glastone, M., Crystal, D., and Schwartz, M. K., *Clinical Chemsitry,* **35** (1989), 1532.
20. Boink, A. B. T. H., Buckley, B. M., Christiansen, T. F., Covington, A. K., Maas, A. H. J., Muller Plathe, O., Sachs, C., and Siggaard-Andersen, O., Recommendations for sampling, transport and storage for the determination of the substance concentrations of ionized calcium. In *Methodology and Clinical Applications of Ion Selective Electrodes. Proceedings of the 8th Meeting of the European Working Group on Ion Selective Electrodes.* Eds Maas, A. H. J., Buckley, B. M., Marsoner, H. J. Saris, N. E. L., and Sprokholt, R. (Graz, 1986, Elinkwijk Printing Company, Utrecht, The Netherlands, 1987), p. 81.
21. Thode, J., Fogh, Andersen, N., Aas, F., and Siggaard-Andersen, O., *Scandinavian Journal of Clinical Laboratory Investigation,* **45** (1985), 131.
22. Gouget, B., Gourmelin, Y., Blanchet, F., Capolaghi, B., Feuillu, A., Lagente, M., Lardet, G., Manceaux, J. C., Pasquier, C., Turret, M., and Truchaud, A. *Annals of Biological Chemistry,* **46** (1988), 419.
23. Siggaard-Andersen, O., and Maas, A. H. J., The IFCC Committee on blood gases and electrolytes and the working group on ion selective electrolytes: past and figure. In *Methodology and Clinical Applications of Ion Selective Electrodes. Proceedings of the 10th Meeting of the European Working Group on Ion Selective Electrodes.* Eds Maas, A. H. J., Buckley, B. M., Marsoner, H. J., Manzoni, A., Moran, R. F., Siggaard-Andersen, O., and Sprokholt, R. (Stresa 1988, Elinkwijk Printing Company, Utrecht, The Netherlands, 1989).

